# PrimerPooler: automated primer pooling to prepare library for targeted sequencing

**DOI:** 10.1093/biomethods/bpz015

**Published:** 2019-11-05

**Authors:** Silas S Brown, Yun-Wen Chen, Ming Wang, Alexandra Clipson, Eguzkine Ochoa, Ming-Qing Du

*Biology Methods and Protocols*, 2017, 2(1), bpx006. doi:10.1093/biomethods/bpx006

The server people.ds.cam.ac.uk has been permanently shut down. The source codes and executables are now available at: http://ssb22.user.srcf.net/pooler/.

The article has been updated to reflect this.

